# Changes in sexual behaviour and practice and HIV prevalence indicators among young people aged 15–24 years in Zambia: An in-depth analysis of the 2001–2002 and 2007 Zambia Demographic and Health Surveys

**DOI:** 10.1080/17290376.2014.903620

**Published:** 2014-04-07

**Authors:** Joshua Kembo

**Affiliations:** PhD, is an Associate Professor in the Bureau of Market Research (BMR) at the University of South Africa (Unisa), South Africa.

**Keywords:** sexual behaviour and practice, young people, Demographic and Health Survey, HIV and AIDS, Zambia, comportement sexuel et les pratiques, les jeunes, Enquête démographique et de santé, le VIH et le SIDA, Zambie

## Abstract

HIV and AIDS still pose a major public health problem to most countries in sub-Saharan Africa, Zambia included. The objective of the paper is to determine changes in selected sexual behaviour and practice and HIV prevalence indicators between 2001–2002 and 2007. We used the Demographic and Health Survey Indicators Database for the computation of the selected indicators. We further used STATA 10.0 to compute significance tests to test for statistical difference in the indicators. The results indicate some changes in sexual behaviour, as indicated by an increase in abstinence, use of condoms and the decrease in multiple partnerships. The overall percentage of abstinence among never-married young men and women aged 15–24 years in Zambia increased significantly by 15.2% (*p* = .000) and 5.9% (*p* = .001) respectively, between 2001–2002 and 2007. A statistically significant increase of 6.6% (*p* = .029) was observed in the percentage of young women who reported having used a condom during the last time they had had premarital sex. A statistically significant decrease of 11.0% (*p* = .000) and 1.4% (*p* = .000) was observed among young men and women, respectively, who reported having multiple partners in the preceding 12 months. The factorial decomposition using multivariate analysis reveals that the indicators which contributed to the statistically significant 2.6% decline in HIV prevalence among young women aged 15–24 years in Zambia include proportion reporting condom use during premarital sex (+6.6%), abstinence (+5.9%), sex before age 15 (– 4.5%), premarital sex (– 2.6%), sex before age 18 (– 2.4%) and proportion reporting multiple partnerships (– 1.4%). Remarkable strides have been achieved towards promoting responsible sexual behaviour and practice among young people in Zambia. Further research focusing on factors that predispose young women in Zambia to higher risk of infection from HIV is required. The results from this paper should be useful in the design of programmes to control the spread of HIV and AIDS, particularly among young people in Zambia and other sub-Saharan countries.

## Résumé

VIH et le SIDA posent encore un problème majeur de santé publique dans la plupart des pays d'Afrique sub-saharienne, la Zambie inclus. L'objectif de l'étude est de déterminer les changements de comportement sexuel choisi et la pratique et les indicateurs de prévalence du VIH entre 2001–02 et 2007. Nous avons utilisé la base de données démographiques et les indicateurs Enquête sur la santé pour le calcul des indicateurs retenus. Nous avons également utilisé STATA 10.0 pour calculer les tests de signification pour tester la différence statistique dans les indicateurs. Les résultats indiquent des changements dans le comportement sexuel comme indiqué par une augmentation de l'abstinence, l'utilisation du préservatif et la diminution de multiples partenariats. Le pourcentage global de l'abstinence chez les jeunes hommes et les femmes n'ayant jamais été mariées âgées de 15–24 ans en Zambie a considérablement augmenté par 15.2% (*p* = .000) et 5.9% (*p* = .001) respectivement, entre 2001–02 et 2007. Une augmentation statistiquement significative de 6.6% (*p* = .029) a été observée dans le pourcentage de jeunes femmes qui ont déclaré avoir utilisé un préservatif lors de la dernière fois qu'ils avaient eu des rapports sexuels avant le mariage. Une diminution statistiquement significative de 11.0% (*p* = .000) et 1.4% (*p* = .000) a été observée chez les jeunes hommes et les femmes, respectivement, qui ont déclaré avoir des partenaires multiples au cours des 12 mois précédents. La décomposition factorielle en utilisant une analyse multivariée montre que les indicateurs qui ont contribué à la baisse statistiquement significative de 2.6% de la prévalence du VIH parmi les jeunes femmes de 15–24 ans en Zambie comprennent proportion déclarant utiliser des préservatifs pendant les rapports sexuels avant le mariage (+6.6%), l'abstinence (+ 5.9%), le sexe avant 15 ans (– 4.5%), le sexe avant le mariage (– 2.6%), le sexe avant 18 ans (– 2.4%) et la proportion de rapports multiples partenariats (– 1.4%). Des progrès remarquables ont été accomplis en vue de promouvoir un comportement sexuel responsable et la pratique chez les jeunes en Zambie. De plus amples recherches en se concentrant sur les facteurs qui prédisposent les jeunes femmes en Zambie à un risque plus élevé d'infection par le VIH est nécessaire. Les résultats de cette étude devraient être utiles dans la conception de programmes de lutte contre la propagation du VIH et du sida, en particulier parmi les jeunes, en Zambie et d'autres pays d'Afrique sub-saharienne.

## Introduction

This paper explores changes in sexual behaviour and practice among young people aged 15–24 years in Zambia. In doing so, the paper draws a comparison between the Zambia Demographic and Health Survey conducted in 2001–2002 (ZDHS 2001–2002) and the ZDHS 2007. The broad objective of the paper is to determine the magnitude and statistical significance of the changes in the selected indicators. The paper offers an in-depth analysis of the ZDHS 2001–2002 and the ZDHS 2007. The results should be useful in the formulation and design of appropriate policies and programmes to control the spread of HIV and AIDS among young people in Zambia and other countries in Southern Africa.

In Zambia, HIV is mostly spread through unprotected heterosexual intercourse (Kalunde 1997). The HIV prevalence in the general population in 2007 in Zambia was 14.3% (Central Statistical Office, Ministry of Health, Tropical Diseases Research Centre, University of Zambia & Macro International Incorporated 2009). Persons living in urban areas had a higher HIV prevalence rate of 19.7% than those residing in rural areas, who had a lower HIV prevalence rate of 10.3% (Central Statistical Office, Ministry of Health, Tropical Diseases Research Centre, University of Zambia & Macro International Incorporated 2009). The HIV prevalence rate among women aged 15–49 years was 16.1%, whereas among men aged 15–59 years the HIV prevalence rate was 12.3%. This places Zambia as a country with one of the highest HIV burdens in Southern Africa (Central Statistical Office, Ministry of Health, Tropical Diseases Research Centre, University of Zambia & Macro International Incorporated 2009).

Young people and children constitute close to 50% of the population in the developing world (United Nations, Department of Economic and Social Affairs, Population Division 2009). AIDS casts a shadow of uncertainty on the livelihoods of young people (Bayles, Bujra, Chabala, Kaihula & Latto-Katundu 1998; Slonin-Nevo & Mukura 2005). Apart from social and economic regressions from the past decades, young people in Africa are also faced with the HIV epidemic. Young Africans have not known a world without HIV and AIDS. Africa encompasses 69% of the world's persons living with HIV yet the continent comprises 14.5% of the world's population (Joint United Nations Programme on HIV/AIDS 2010a).

Family values and norms describing sexual behaviour have changed to a great extent in many African countries, including Zambia (Joint United Nations Programme on HIV/AIDS 2010a). Sex education is limited in schools in Zambia; young persons have to learn about sexual education from their peers. There is therefore increased risk of sexually transmitted infections and teenage pregnancy among young people (Joint United Nations Programme on HIV/AIDS 2010a).

Sexually transmitted infections are a major public health concern among young people in Zambia. According to Ndubani (2002), young people are the most affected by sexually transmitted infections and close to 200,000 cases involving sexually transmitted infections are treated at formal health facilities in Zambia annually (Ndubani 2002). The major cause of the spread of sexually transmitted infections among young persons in Zambia is high-risk sexual behaviour and practice. High-risk sexual behaviour refers to sexual behaviour that promotes the spread of sexually transmitted infections through unprotected sex and multiple sexual partnerships (Mukelabai & Imasiku 2005; Ndubani 2002; Ndubani & Hojer 2001). High-risk sexual behaviour exposes young men and women to infection from HIV. In the rural study district, Chiawa, condoms are perceived to affect male potency (Mukelabai & Imasiku 2005; Ndubani 2002; Ndubani & Hojer 2001). Multiple sexual partnerships and unprotected sex are reported to be rampant among the young men living in Chiawa district of Zambia (Mukelabai & Imasiku 2005; Ndubani 2002; Ndubani & Hojer 2001). Perceptions of manhood were observed as a major factor that causes multiple sexual relationships among young people in Zambia (Ndubani 2002).

Most HIV infections are occurring among young people aged between 15 and 24 years in Africa (Ndubani 2002). A number of young people, particularly those in their teens are reportedly underrepresented among those who are accessing voluntary counselling and testing (VCT) services in Zambia (Denison, Sweat, Dunnett-Dagg, Lungu & McCauley 2005). A further observation is that among young people, HIV is most common among young women. This observed gender disparity warrants further investigation with regard to the social and cultural factors that support and sustain risky sexual behaviour (Denison *et al.* 2005).

Comprising 30% of the population in the developing countries, young people present social and economic challenges that are the key to the stability of the country. Communities are urged to give young people opportunities to play a role in politics, social mobilisation and the economy. In Zambia and Uganda, young people have been instrumental in reducing the HIV prevalence in these countries through embracing more responsible behaviour (Denison *et al.* 2005). Moreover, young people have the potential to play a vital role in slowing down the spread of the HIV and AIDS epidemic in Africa (Carrino 2005).

Sexual behaviours among young people are, to a large extent, influenced by individual desires, and environmental and economic processes (Roach & Wisman 2005). This adds a complexity to programmes and initiatives for the prevention of HIV and AIDS. In order to move forward, a holistic approach is required that addresses specific behaviour patterns and introduces a package for an appropriate response. Improving the livelihoods of young people in Zambia, and indeed in other African settings, is fundamental in persuading them to adopt more positive health-seeking behaviours (Kimuna & Djamba 2005; Mukelabai & Imasiku 2005; Roach & Wisman 2005).

A study conducted in Kenya, Uganda and Zambia shows that social relationships influence young persons’ decisions pertaining to whether they agree to undergo testing for HIV (Lee 2006). It is further observed that families play a vital role in a young person's decision-making process to test for HIV. The study underlines the importance of taking into cognisance societal norms and values, including social interaction in the design of HIV and AIDS prevention programmes for young people (Lee 2006; Roach & Wisman 2005).

In another study in Zambia, the researchers conclude that young people that have attended sexuality and skills programmes designed to persuade them to abstain from sex until marriage (abstinence, behaviour change, youth) reject inaccurate information and demand condoms. The researchers argue that efforts should be strengthened to reduce negative messages about the use of condoms if they are to be acceptable, particularly among young people for the prevention of sexually transmitted infections, including HIV and AIDS (International HIV/AIDS Alliance 2007).

In a study that involved studying the effect that viewing a risk-reduction media campaign has on reducing the risk of HIV transmission among adolescents in Zambia, the researchers observe an elevated risk ratio of 2:1 among adolescents who had watched at least three television spot advertisements of the helping each other act responsibly together (HEART) campaign compared with their counterparts who reported to the contrary. The results further indicate that the HEART campaign viewers were 2.4 times more likely to have ever used a condom and 1.6 times more likely to report primary or secondary abstinence, compared to those who had not viewed the campaign (Gordon & Mwale 2006; Underwood, Hachonda, Serlemitsos & Bharath-Kumar 2006).

In another study in Zambia, the researchers conclude that HIV prevalence among young people, particularly in the higher education categories declined between 1997 and 2003 (Sandoy, Michelo, Siziya & Fylkesnes 2007). The authors further note that, overall, high-risk sexual behaviour decreased, condom use increased, the percentage of young people with multiple sexual partners decreased and that there were substantial delays in the age at which young women gave birth (Sandoy *et al.* 2007).

There are efforts underway to reduce the spread of HIV infection among young people in Zambia. A case in point is the Youth Peer Education Programme (YPE), which is a widely used strategy to promote sexual and reproductive health and reduce the spread of HIV and AIDS. In this regard, exposure to YPE in Zambia led to sexual and reproductive health risk-reduction behaviours among young people. A major challenge cited is that the YPE should be scaled upwards so as to be able to reach as many vulnerable young people in Zambia as possible (Stevenson, Burke & Johnson 2008).

The importance of the provision of accurate information as a vehicle that facilitates the prevention of HIV infection is supported by the International HIV/AIDS Alliance (International HIV/AIDS Alliance 2007). The provision of accurate information to young people helps them to protect themselves from sickness so that they can grow up happy and healthy (International HIV/AIDS Alliance 2007).

Zambia is one of the countries in the Southern African region most affected by the HIV and AIDS epidemic, drastically impacting on young people (International HIV/AIDS Alliance 2007). Several perceptions related to socio-economic and cultural factors prevent young people from changing their sexual behaviour despite awareness of the risks associated with multiple sexual partnerships (International HIV/AIDS Alliance 2007). Positive change in sexual behaviour and practice among young people is fundamental in the control of the spread of HIV and AIDs in Zambia (International HIV/AIDS Alliance 2007). According to the Sexual Behaviour Survey conducted in Zambia in 2009, the percentage of young women and men aged 15–24 years who have had sexual intercourse before the age of 15 years was 6.8% and 8.2%, respectively (Central Statistical Office, Ministry of Health, University of Zambia & MEASURE Evaluation 2010). The percentage of young persons who reported condom use during their last sex was 37%. Approximately 50% of the young surveyed females (50%) reported having had a pregnancy. This result is particularly worrying because unprotected sex potentially exposes young people to sexually transmitted infections, including HIV and AIDS. Similar surveys were conducted in 2000 and 2003 in Zambia (Central Statistical Office, Ministry of Health, University of Zambia & MEASURE Evaluation 2010).

Young people are at the centre of the HIV prevention revolution, particularly in Africa. HIV prevalence is declining in 16 of the 25 countries most affected by the epidemic (Gouws 2010). The major driver of the decline of HIV infection in these 16 countries is behaviour change, particularly among young people who are choosing to have fewer sexual partners (Joint United Nations Programme on HIV/AIDS 2010b). Zambia is one of the three countries that have shown declines in the HIV prevalence among young women in national population surveys, the other two being South Africa and Tanzania, which showed declines in HIV prevalence among young men (Gouws 2010). There are changing factors that influence sexual behaviour among young people in Zambia. Primary schools should be focal points in imparting sex education to young people. This will enhance safer sexual behaviour, thereby mitigating the risk of sexually transmitted infections, including HIV infection (Gordon 2008).

Neighbourhood education is a strong determinant of HIV infection among young women aged 15–24 years in both rural and urban areas in Zambia (Kayeyi, Sandoy & Fylkesnes 2009). According to Poundstone, Strathdee and Celentano (2004), neighbourhood factors include social networks and physical places through which diseases spread. The authors argue that studying the individual-level determinants of health outcomes alone results in a limited understanding of the dynamics of health outcomes. Women living in rural and urban areas in Zambia are associated with 3.4 times and 1.8 times greater risk of HIV infection, respectively, after adjusting for age and individual level of neighbourhood education and variables such as education (Kayeyi *et al.* 2009). After controlling for the level of neighbourhood education, there is an observed protective effect, particularly among women living in urban areas. It therefore appears that neighbourhood education has a protective effect against risk of HIV infection, more so among women living in urban areas of Zambia.

HIV-related stigma remains a major challenge in the control of HIV infection among young people. In a study conducted among persons aged 15–24 years old in Burkina Faso, Ghana and Zambia, the researchers found that economic and behavioural aspects of the young persons were strongly associated with HIV-related stigma. The researchers concluded that if behavioural change interventions are to be effective in the control of HIV and AIDS, they should incorporate HIV-related stigma. Such interventions should be sensitive to and consider the cultural, social and economic dimensions among different populations (Stephenson 2009).

## Data and methods

This paper used data from the 2001–2002 and 2007 ZDHSs. The Demographic and Health Survey (DHS) is a population-based and nationally representative retrospective survey conducted in most countries every five years. A DHS consists of a series of cross-sectional surveys, designed using a multi-stage stratified sample design. The sample is stratified by province, ward, enumeration area, cluster and household. A DHS also uses standard questionnaires with independently selected samples in each round of the survey. The 2001–2002 and 2007 ZDHSs were designed and implemented with technical assistance from Macro International Incorporated.

A DHS was first conducted in Zambia in 1992. The 2001–2002 and 2007 ZDHS were the third and fourth, respectively, to be conducted in Zambia. The ZDHSs are part of the worldwide DHS project. These surveys are designed to collect data on maternal and child health, fertility, mortality, family planning, nutrition and HIV and AIDS.

The 2001–2002 ZDHS was implemented by the Zambia Central Statistical Office (CSO) and the Zambia Central Board of Health between November 2001 and May 2002. The sample included 7126 households, 7658 women aged 15–49 and 2145 men aged 15–59. The survey results are representative at the national level, by urban and rural residence, and nine provinces (Central Statistical Office, Central Board of Health & Macro International Incorporated 2003).

The 2007 ZDHS was also implemented by the CSO in partnership with the Ministry of Health in Zambia from April to October 2007. A nationally representative sample of 7164 households, 7146 women aged 15–49 and 6500 men aged 15–49 were interviewed. The 2007 ZDHS is a follow-up to the 1992, 1996, and 2001–2002 ZDHSs and provides updated estimates of basic demographic and health indicators covered in the earlier surveys. The 2007 ZDHS is the second survey in Zambia to provide population-based prevalence estimates for HIV (Central Statistical Office, Ministry of Health, Tropical Diseases Research Centre, University of Zambia & Macro International Incorporated 2009).

This paper utilised the HIV/AIDS Survey Indicators Database to extract the selected young people's sexual behaviour and practice and HIV prevalence indicators from the 2001–2002 and 2007 ZDHSs. The percentage differences in the indicators between 2001–2002 and 2007 were computed using the STATCOMPILER programme available in the HIV/AIDS Survey Indicators Database. The HIV/AIDS Survey Indicators Database uses a core schedule of indicators adopted jointly by the Joint United Nations Programme on HIV/AIDS (UNAIDS) and the United States Agency for International Development in 2000. The indicators were designed to monitor the goals as articulated at the United Nations General Assembly Special Session on HIV/AIDS and the millennium development goals. The indicators from the HIV/AIDS Survey Database can be reported either cross-sectionally or using a time line series.

In order to determine any demographic variations by sex and area of residence, the data for the indicators were tabulated by sex and urban/rural residence. The significance tests of statistical difference in the indicators between 2001–2002 and 2007 were computed using STATA 10.0 software (Stata Corporation 2003). The significance tests were performed at the level of *p* = .005.

Ethical considerations are well catered for in all the DHSs around the world. In the 2001–2002 and 2007 ZDHSs, informed written consent was sought from the respondents before commencing the interview (Central Statistical Office, Ministry of Health, Tropical Diseases Research Centre, University of Zambia & Macro International Incorporated 2009). A general health schedule was administered before collecting blood samples. The respondents were further required to provide written informed consent before undertaking the dried blood sample HIV sero-status test (Central Statistical Office, Ministry of Health, Tropical Diseases Research Centre, University of Zambia & Macro International Incorporated 2009). The cluster and household identification codes were removed from the respondent's questionnaire in order to ensure anonymity of the blood sample. The survey participants were further provided with information on the availability of VCT facilities around Zambia (Central Statistical Office, Ministry of Health, Tropical Diseases Research Centre, University of Zambia & Macro International Incorporated 2009).

## Results

We in this section first present the profile of young people in Zambia based on the Zambia 2007 DHS. We then present the results based on the changes in the sexual and behaviour indicators. Finally, we present the multivariate analysis of the changes in the indicators in order to show the impact of the observed changes on programmes and policies towards the control of HIV infection in Zambia.

## Profile of young people in Zambia

The population of Zambia as enumerated in the 2010 population and housing census was 13,092,666 persons. This represents an increase of 32.4% from the population of 9,885,591 as enumerated in 2000. The population of Zambia is projected to increase to 15.5 million by 2015 at an annual growth rate of 2.4%. At this rate, the population is expected to double by 2030 (Central Statistical Office 2001). Young people aged 15–24 constitute 20.8% of the total population of Zambia (Central Statistical Office 2012).

Apart from being socially, economically and politically alienated from the mainstream of national development, young people in Zambia are faced with a huge HIV and AIDS epidemic (Government of the Republic of Zambia 2006). Youth programmes in Zambia are also hindered by a myriad of factors that include poor information flow, poor access to information for young people, especially in rural areas, and limited participation of young people in decision-making processes. This has inadvertently hindered programmes targeted at alleviating the spread of HIV, including STIs among young people in Zambia.

## Changes in the indicators

The results on the changes in selected sexual behaviour and practice and HIV indicators among young people aged 15–24 years from the 2001–2002 ZDHS and 2007 ZDHS are presented below.

The first indicator, presented in Table 1 deals with abstinence among never-married young men and women aged 15–24 years. This indicator refers to the percentage of never-married young women and men aged 15–24 who have never had sex. The results presented in Table 1 indicate that overall in Zambia the percentage of abstinence among never-married young men and women aged 15–24 years increased significantly by 15.2% (*p* = .000) and 5.9% (*p* = .001), respectively, between 2001–2002 and 2007. A comparison by area of residence reveals that this increase was only significant among young persons aged 15–24 years residing in urban areas as compared to their counterparts who lived in rural areas. The percentage of abstinence among never-married young men and women aged 15–24 years who resided in urban areas increased significantly by 27.1% (*p* = .000) and 9.3% (*p* = .000), respectively, from 2001–2002 to 2007.

**Table 1.** Abstinence of never-married young men and women aged 15–24, Zambia DHS 2001–2002 and Zambia DHS 2007.

					Residence							
	Total				Urban				Rural			
	Sex											
	Men		Women		Men		Women		Men		Women	
	Indicator value %											
2001–2002	28.2	688	50.3	1732	19.3	292	50.0	860	34.9	396	50.6	872
2007	43.4	2155	56.2	1629	46.4	1087	59.3	906	40.3	1068	52.3	723
*p*-Value	.000		.001		.000		.000		.060		.499	

*p* < .05.

Source: All tables based on author computations using the online STATCOMPILER available from the HIV/AIDS Survey Indicators Database obtainable from the following Measure DHS Website: http://hivdata.measuredhs.com/data/start.cfm?survey_type_id=&survey_pop_based=&action=new_table&userid=99268$usertabid=113580CFID=1799857&CFTOKEN=53855275.

The data on the percentage of young people aged 15–24 years who had had sex before the age of 15 years based on the 2001–2002 and 2007 ZDHSs are presented in Table 2. Overall, the percentage of young people who had had sex before the age of 15 years declined between 2001–2002 and 2007. There is statistically significant difference in the overall percentage of young men and women aged 15–24 years who reported that they had had sex before the age of 15 years between 2001–2002 and 2007. Overall, there was a significant decline of 9.8% (*p* = .000) and 4.4% (*p* = .000) among young men and women aged 15–24 years respectively, in the percentage that had had sex before the age of 15 years. Young men and women living in urban areas in Zambia experienced a significant decline of 14.1% (*p* = .000) and 4.7% (*p* = .000) in the percentage that had had sex before the age of 15 years, respectively. Their counterparts residing in rural areas were associated with significant declines of 6.5% (*p* = .002) and 3.8% (*p* = .004), respectively.

**Table 2.** Sex before the age of 15 years among young men and women aged 15–24, Zambia DHS 2001–2002 and Zambia DHS 2007.

					Residence							
	Total				Urban				Rural			
	Sex											
	Men		Women		Men		Women		Men		Women	
	Indicator value %											
2001–2002	25.8	804	17.9	3476	28.9	330	14.2	1457	23.6	475	20.6	2019
2007	16.0	2482	13.5	2944	14.8	1187	9.5	1352	17.1	1296	16.8	1592
*p*-Value	.000		.000		.000		.000		.002		.004	

*p* < .05.

Table 3 is an extension of Table 2 and provides the results on the percentage of young people who had had sex before the age of 18 years as observed in the 2001–2002 and 2007 ZDHSs. There was an overall significant decline of 8.2% (*p* = .000) among young men aged 15–24 who reported that they had had sex before the age of 18 years. The analysis by area of residence reveals that the decline of 9.4% among young men aged 15–24 years residing in urban areas is significant (*p* = .035). Although there was a reduction in the overall percentage among young women aged 15–24 years who reported having sex before the age of 18 years between 2001–2002 and 2007, the decrease is non-significant.

**Table 3.** Sex before age 18 years among young men and women aged 15–24, Zambia DHS 2001–2002 and Zambia DHS 2007.

					Residence							
	Total				Urban				Rural			
	Sex											
	Men		Women		Men		Women		Men		Women	
	%	*n*	%	*n*%	%	*n*%	%	*n*	%	*n*	%	*n*
2001–2002	58.8	346	62.2	1664	55.7	166	51.8	693	61.8	180	69.6	971
2007	50.6	1066	59.8	1370	46.3	508	48.1	590	54.5	559	68.6	780
*p*-Value	.008		.177		.035		.186		.086		.652	

*p* < .05.

Table 4 provides the results on primary abstinence. This indicator refers to the percentage of young women and men aged 15–19 years who had never had sex. Data on this indicator were collected only from the 2007 ZDHS. Overall, 55.3% and 51.9% of young men and women aged 15–19 years respectively, experienced primary abstinence (*p* = .063). This difference is statistically not significant. Primary abstinence was significantly higher among young men living in the rural areas relative to young women residing in the same area, that is, 51.2% and 44.2%, respectively (*p* = .006).

**Table 4.** Primary abstinence among young men and women aged 15–24, Zambia DHS 2007.

					Residence							
	Total				Urban				Rural			
	Sex											
	Men		Women		Men		Women		Men		Women	
	%	*n*	%	*n*%	%	*n*%	%	*n*	%	*n*	%	*n*
2007	55.3	1416	51.9	1574	59.7	679	60.2	761	51.2	737	44.2	813

Note: Data available for Zambia DHS 2007 only.

Table 5 provides data on secondary abstinence. This indicator refers to the percentage of young women and men aged 15–24 years who had not had sex in the last year of those who ever had sex. Data for this indicator were collected only for the 2007 ZDHS. The overall percentage of young men who reported secondary abstinence in the 2007 ZDHS is significantly higher than that of young women, 27.2% compared to 14.5%, respectively (*p* = .000). In urban and rural areas, separately, secondary abstinence is also significantly higher among young men than young women. The percentage of young men who reported secondary abstinence in urban areas was 35.2% compared to 18.1% for their young female counterparts (*p* = .000). Similarly, the percentage of young men aged 15–24 years living in rural areas who reported experiencing secondary abstinence was 20.9% compared to 12.0% for their female counterparts (*p* = .000).

**Table 5.** Secondary abstinence among young men and women aged 15–24, Zambia DHS 2007.

					Residence							
	Total				Urban				Rural			
	Sex											
	Men		Women		Men		Women		Men		Women	
	%	*n*	%	*n*%	%	*n*%	%	*n*	%	*n*	%	*n*
2007	27.2	1548	14.5	2029	35.2	683	18.1	814	20.9	866	12.0	1214

Note: Data available for Zambia DHS 2007 only.

Table 6 provides the results for the indicator dealing with premarital sex in the last year before the survey. This indicator refers to the percentage of young never-married people aged 15–24 years who had had sex in the 12 months before the survey of all young single people surveyed in the 2001–2002 and 2007 ZDHSs, respectively. The results indicate that overall there were reductions in the percentages of young people who had had premarital sex in the 12 months before the 2001–2002 ZDHS and 2007 ZDHS. However, the decreases are only statistically significant for young men. Overall, there is a statistically significant decrease of 13.7% (*p* = .000) from 2001–2002 to 2007 in the percentage of young men aged 15–24 years who had had premarital sex in the 12 months before the survey. The decrease is more pronounced among young men than among their female counterparts, for those living in urban areas. Young men aged 15–24 years who resided in urban areas are associated with a statistically significant decrease of 18.2% (*p* = .000) in the percentage who reported that they had had premarital sex in the 12 months before the 2001–2002 and 2007 ZDHSs. Young men aged 15–24 years who lived in the rural areas are associated with a statistically significant decrease of 8.9% (*p* = .002).

**Table 6.** Young men and women aged 15–24 having premarital sex in the year before the surveys, Zambia DHSs 2001–2002 and 2007.

					Residence							
	Total				Urban				Rural			
	Sex											
	Men		Women		Men		Women		Men		Women	
	%	*n*	%	*n*%	%	*n*%	%	*n*	%	*n*	%	*n*
2001–2002	51.3	688	31.3	1732	50.3	292	28.4	860	52.1	396	34.2	872
2007	37.6	2155	28.7	1629	32.1	1087	26.0	906	43.2	1068	32.1	723
*p*-Value	.000		.100		.000		.257		.002		.375	

*p* < .05.

Table 7 presents results on young men and women aged 15–24 years who reported using a condom during premarital sex in the 12 months before the 2001–2002 and 2007 ZDHSs. This indicator refers to the percentage of young never-married people (aged 15–24 years) who used a condom at last sex, of all young single sexually active people surveyed. Overall, there is a statistically significant increase of 6.6% (*p* = .029) in the percentage of young women who reported having used a condom during the last time they had premarital sex. Although there are increases in the percentage of young men who reported using a condom during premarital sex between 2001–2002 and 2007 ZDHSs, these increases are statistically insignificant.

**Table 7.** Young men and women aged 15–24 using a condom during premarital sex, Zambia DHSs 2001–2002 and 2007.

					Residence							
	Total				Urban				Rural			
	Sex											
	Men		Women		Men		Women		Men		Women	
	%	*n*	%	*n*%	%	*n*%	%	*n*	%	*n*	%	*n*
2001–2002	41.1	353	32.4	542	49.6	147	42.3	244	35.1	206	24.2	298
2007	47.3	810	39.0	468	56.2	348	51.8	236	40.7	461	25.9	232
*p*-Value	.051		.029		.178		.037		.171		.654	

*p* < .05.

Table 8 provides the results on young people having multiple partners in the 12 months before the 2001–2002 and 2007 ZDHSs. This indicator specifically refers to the percentage of young people (aged 15–24 years) who had had sex with more than one partner in the 12 months preceding the ZDHSs, of all sexually active young people. Overall, there is a statistically significant decrease of 11.0% (*p* = .000) and 1.4% (*p* = .000) among young men and women, respectively, who reported having multiple partners in the 12 months before the 2001–2002 and 2007 ZDHSs. The analysis by area of residence also reveals that the decreases are statistically significant. For young men and women who resided in urban areas, there is a statistically significant decrease of 9.1% (*p* = .000) and 2.1% (*p* = .000), respectively. A similar observation is made for their counterparts living in rural areas of Zambia. There is a statistically significant decrease in the percentage who reported having had multiple partners in the year before the 2001 and 2007 ZDHSs among young men and women residing in rural areas, 12.2% (*p* = .000) and 1.1% (*p* = .000), respectively.

**Table 8.** Young men and women aged 15–24 having multiple partners in the year before the surveys, Zambia DHSs 2001–2002 and 2007.

					Residence							
	Total				Urban				Rural			
	Sex											
	Men		Women		Men		Women		Men		Women	
	%	*n*	%	*n*%	%	*n*%	%	*n*	%	*n*	%	*n*
2001–2002	30.3	467	3.9	2150	30.6	184	4.7	796	30.1	283	3.5	1354
2007	19.3	1127	2.5	1735	21.5	442	2.6	667	17.9	685	2.4	1068
*p*-Value	.000		.000		.015		.000		.000		.000	

*p* < .05.

The results on condom use at first sex are presented in Table 9. This indicator depicts the percentage of young people (aged 15–24 years) who had used a condom the first time they ever had sex, of those who had ever had sex. Data on condom use at first sex among young men and women aged 15–24 years in Zambia were collected only in the 2007 ZDHS. The results in Table 9 indicate that, overall, about a fifth of young men (21.6%) and nearly a quarter of young women (24.1%) had used a condom the first time they ever had sex (*p* = .078). This sex differential is, however, statistically non-significant. A comparison of condom use among young persons aged 15–24 years living in urban and rural areas reveals that in urban areas young women are significantly more likely to have used a condom during the first time they had sex than their female counterparts, 36.8% and 25.7%, respectively (*p* = .000). The converse is true with regard to rural areas. In rural areas, young men aged 15–24 years are more likely to have used a condom the first time they had sex (18.4%) than their female counterparts (15.6%) (*p* = .092). The difference between young men and women is also statistically insignificant.

**Table 9.** Condom use at first sex among young women and men aged 15–24, Zambia DHS 2007.

					Residence							
	Total				Urban				Rural			
	Sex											
	Men		Women		Men		Women		Men		Women	
	%	*n*	%	*n*%	%	*n*%	%	*n*	%	*n*	%	*n*
2007	2.6	1548	24.1	2029	25.7	683	36.8	814	18.4	866	15.6	1214

Note: Data available for Zambia DHS 2007 only.

Table 10 provides results on age-mixing among young women aged 15–24 years in Zambia. This indicator refers to the percentage of young women aged 15–24 years who had had sex in the preceding 12 months with a partner who is 10 or more years older than themselves. Data on age-mixing was collected only for the 2007 ZDHS. The results in Table 10 indicate that 2.2% of young women aged 15–24 years who had had sex in the preceding 12 months had had sex with a partner who was 10 or more years older than themselves. The percentage of young women who practised age-mixing is significantly higher in urban (3.6%) than in rural areas (1.4%) *(p* = .003).

**Table 10.** Age-mixing in sexual partnerships (young women aged 15–24, any partner) in the 12 months before Zambia DHS 2007.

			Residence			
	Total		Urban		Rural	
	%	*n*	%	*n*%	%	*n*%
2007	2.2	1735	3.6	667	1.4	1068

Note: Data available for Zambia DHS 2007 only.

Table 11 provides data on sex among young people while they are intoxicated. This indicator refers specifically to the percentage of young people who had had sex while they or their partners were intoxicated, during the preceding 12 months. The data for this indicator in Zambia were collected only in the 2007 ZDHS. Overall, a significantly higher percentage of young women (7.8%) than young men (4.9%) had had sex while they or their partners were intoxicated, during the 12 months preceding the 2007 ZDHS (*p* = .000). The percentage of young people residing in urban areas who had had sex while they or their partners were intoxicated is significantly higher among young women than among young men, that is 8.1% and 6.9%, respectively *(p* = .000). A similar pattern is observed among young people living in rural areas. The percentage of young persons who had had sex while they or their partners were intoxicated is significantly higher for young women (7.5%) than for young men (3.0%) *(p* = .000).

**Table 11.** Sex among young people aged 15–24 while they are intoxicated, Zambia DHS 2007.

					Residence							
	Total				Urban				Rural			
	Sex											
	Men		Women		Men		Women		Men		Women	
	%	*n*	%	*n*%	%	*n*%	%	*n*	%	*n*	%	*n*
2007	4.9	2482	7.8	2944	6.9	1187	8.1	1352	3.0	1296	7.5	1592

Note: Data available for Zambia DHS 2007 only.

Table 12 provides results on sex with commercial sex workers (CSWs) among young men aged 15–24 years. This indicator refers to the percentage of young men who had had sex with a CSW in the preceding 12 months. The data for this indicator were collected only in the 2007 ZDHS. An overall 5.0% of young men aged 15 – 24 years had had sex with a CSW in the 12 months prior to the 2007 ZDHS. A significantly higher percentage of young men aged 15 – 24 years living in rural (6.9%) than those residing in urban areas (3.0%) reported that they had had sex with a CSW in the 12 months before the survey *(p* = .000).

**Table 12.** Sex with CSWs among young people aged 15–24, Zambia DHS 2007.

			Residence			
	Total		Urban		Rural	
	%	*n*	%	*n*%	%	*n*%
2007	5.0	2482	3.0	1187	6.9	1296

Note: Data available for Zambia DHS 2007 only

Table 13 provides results on the changes in HIV prevalence rates among young men and women aged 15 – 24 years between 2001–2002 and 2007 ZDHSs. This indicator refers to the percentage of young people aged 15–24 years who are HIV infected. Overall, there is a statistically significant increase in HIV prevalence of 1.3% *(p* = .000) among young men aged 15–24 years between 2001 – 2002 and 2007 in Zambia. Conversely, overall, there is a statistically significant decrease of 2.6% (*p* = .000) in HIV prevalence among young women between 2001–2002 and 2007 in Zambia. A comparison among young men living in urban and rural areas of Zambia reveals that only in the case of young men living in urban areas is the increase in HIV prevalence between 2001–2002 and 2007 statistically significant, that is 2.0% (*p* = .000). With regard to young women living in urban and rural areas, there are statistically significant decreases in HIV prevalence in both cases between 2001–2002 and 2007. For young women aged 15–24 years living in urban and rural areas, HIV prevalence decreased significantly by 4.1% (*p* = .034) and 2.0% (*p* = .000), respectively. The sex differentials in HIV prevalence reveal that in 2001–2002 the overall estimate for young women aged 15–24 years (11.1%) is significantly higher than that for their male counterparts (3.0%) (*p* = .000). A similar observation is derived with regard to the HIV prevalence estimates for 2007. In 2007, the overall HIV prevalence for young women aged 15–24 years (8.5%) is significantly higher than that for their male counterparts (4.3%) (*p* = .000).

**Table 13.** HIV prevalence rates among young men and women aged 15–24, Zambia DHSs 2001–2002 and 2007.

					Residence							
	Total				Urban				Rural			
	Sex											
	Men		Women		Men		Women		Men		Women	
	%	*n*	%	*n*%	%	*n*%	%	*n*	%	*n*	%	*n*
2001–2002	3.0	675	11.1	941	3.7	275	15.2	395	2.6	400	8.2	546
2007	4.3	2032	8.5	2225	5.7	993	11.1	1032	2.9	1039	6.2	1193
*p-* Value	.000		.000		.000		.034		.257		.000	

*p* < .05.

It is further imperative to analyse changes in HIV prevalence in the general population between 2001–2002 and 2007 in Zambia. HIV prevalence estimates for the general population in Zambia between 2001–2002 and 2007 are provided in Table 14. Overall, there are statistically insignificant decreases in HIV prevalence rates in the general population in Zambia between 2001–2002 and 2007. An analysis with regard to area of residence, urban or rural, reveals a fairly similar pattern. The only statistically significant decrease in HIV prevalence is that observed for the population residing in urban areas of Zambia, that is, 3.3% (*p* = .043). Similar to earlier observations among young persons aged 15–24 years, the HIV prevalence estimates for women in the general population are significantly higher than those for their male counterparts. In 2001–2002, the overall HIV prevalence estimate in the general population for women aged 15–49 years (17.8%) is significantly higher than that for men aged 15–59 years (12.6%) (*p* = .000). In 2007, the HIV prevalence estimate for women aged 15–49 years (16.1%) is also significantly higher than that for men aged 15–59 years (12.3%) (*p* = .000). This observation of higher HIV prevalence rates among women than men in Zambia will be revisited in the following discussion.

**Table 14.** HIV prevalence rates among the general population, Zambia DHSs 2001–2002 and 2007.

					Residence							
	Total				Urban				Rural			
	Sex											
	Men		Women		Men		Women		Men		Women	
	%	*n*	%	*n*%	%	*n*%	%	*n*	%	*n*	%	*n*
2001–2002	12.6	1877	17.8	2073	19.2	676	26.3	808	8.9	1058	12.4	1265
2007	12.3	5374	16.1	5502	15.9	2322	23.1	2317	9.5	3053	11.0	3185
*p*-Value	.734		.076		.043		.066		.563		.185	

*p* < .05.

## Multivariate analysis

**Fig. 1.**
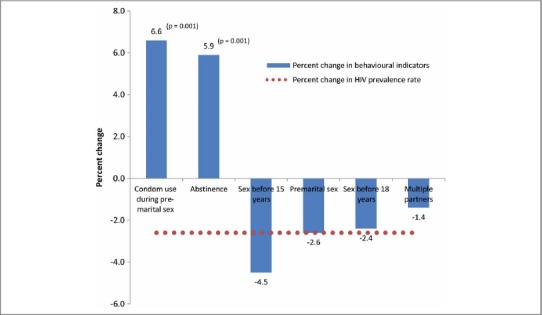
Multivariate analysis of the indicators for sexual behaviour and HIV prevalence among young women aged 15–24, Zambia DHS 2001–2002 and Zambia DHS 2007.

In an earlier section, we showed that the HIV prevalence rate among young women aged 15–24 years in Zambia experienced a statistically significant decline of 2.6% between 2001–2002 and 2007. This section presents results on the multivariate analysis of the indicators for sexual behaviour and HIV prevalence for young women aged 15–24 years, as shown in Fig. 1. The multivariate analysis shows which of the indicators contributed to the observed 2.6% decline in HIV prevalence among young women aged 15–24 years. The results reveal that the indicators that contributed to the statistically significant 2.6% decline in HIV prevalence among young women aged 15–24 years in Zambia include proportion reporting condom use during premarital sex (+6.6%), abstinence (+ 5.9%), sex before age 15 (–4.5%), premarital sex (– 2.6%), sex before age 18 (– 2.4%) and proportion reporting multiple partnerships (– 1.4%). The implications of these results will be revisited for programmatic interventions for the control of HIV and AIDS among young people in the conclusion section of this paper.

## Discussion

This study used two nationally representative surveys, that is the ZDHSs conducted in 2001–2002 and 2007. The study focused on changes in selected indicators dealing with sexual behaviour and practice and HIV prevalence in Zambia between 2001 and 2007. These surveys were the third and fourth to be conducted in Zambia, respectively. Earlier surveys were conducted in Zambia in 1992 and 1996.

The results from this study indicate that programmes dealing with HIV and AIDS in Zambia are having a positive impact on behaviour change among young persons aged 15–24 years. The following six observations can be cited as evidence for this programme impact.

First, the percentage of abstinence among never-married young women and men increased significantly between 2001–2002 and 2007. Second, the percentage of young women and men who had had sex before the age of 15 years decreased significantly between the two survey periods. A similar observation was obtained for young people who had had sex before the age of 18 years, although the decline was only significant among young men. Although data on primary abstinence were collected only in the 2007 ZDHS, this indicator was included in this analysis. It also appears that the impact on behaviour change and practice is being felt more among young persons living in urban areas as compared with their counterparts residing in rural areas.

Third, there was a reduction in the percentage of young never-married people who had had sex in the 12 months before the survey. This decline was more pronounced in the case of young men than their female counterparts across urban and rural areas.

Fourth, there were increases in the percentage of young men and women who reported using a condom during premarital sex in the 12 months before the survey. It was further noted that these increases in condom use are only statistically significant for young women.

Fifth, there were pronounced decreases in the percentage of young men and women, of all sexually active young people, who had had sex with more than one partner in the 12 months preceding the survey. This observation is true for the overall percentages and across the rural and urban sectors as well. Data on the percentage of young people (aged 15–24 years) who used a condom the first time they ever had sex, of those who had ever had sex were collected only for the 2007 ZDHS. Overall, a fifth and a quarter of young men and women aged 15–24 years, respectively, had used a condom the first time they had ever had sex. Young women residing in urban areas were more likely to have used a condom than their male counterparts, whereas the converse was true for those living in rural areas.

Sixth, data on age-mixing revealed that less than 3% of young women had had sex with a person who was 10 or more years older than themselves. This percentage was twice as high in urban compared to rural areas. Similarly, data on the percentage of young people who had had sex while they were intoxicated were collected only during the 2007 ZDHS.

Among the less desirable results is the observation that nearly a tenth of young women aged 15–24 years had sex while they or their partners were intoxicated during the 12 months preceding the 2007 ZDHS. The percentages of young men and woman who had had sex while they or their partners were intoxicated were relatively higher for those living in urban compared to rural areas. Data on sex with CSWs were also collected only during the 2007 ZDHS. An overall 5.0% of young men aged 15–24 years had had sex with a CSW during the 12 months before the 2007 ZDHS. This percentage was relatively higher among young men aged 15–24 years living in urban compared to rural areas.

It further appears that young women aged 15–24 years in Zambia have relatively higher HIV prevalence rates than their male counterparts. An analysis by rural or urban area of residence further confirmed this observation. However, whereas HIV prevalence among young men aged 15–24 years increased significantly between 2001–2002 and 2007, it decreased significantly among their female counterparts. The observed increase in HIV prevalence among young men aged 15–24 years was more pronounced in urban than rural areas. There was a statistically insignificant decline in HIV prevalence in Zambia in the general population among women and men between 2001–2002 and 2007. The results also confirmed that HIV prevalence for women in the general population in Zambia is significantly higher than that for men. Literature has also confirmed that generally women are at a greater risk of heterosexual transmission of HIV. Women are also more likely to be subjected to unprotected heterosexual intercourse than men, are less likely able to negotiate condom use and are more likely to be subjected to non-consensual sex. In 2010, the UNAIDS also reported that approximately three quarters of women living with HIV and AIDS were in sub-Saharan Africa (Joint United Nations Programme on HIV/AIDS 2010a).

## Conclusion and programmatic implications of the findings

Important strides have been taken towards promoting responsible sexual behaviour and practice, including the prevention of HIV and AIDS in Zambia. The results in this paper are testimony to that observation. One limitation of this paper is that some of the observed changes in the indicators between 2001–2002 and 2007 may be due to bias in the reporting of sexual behaviour and practice information among the surveyed young women and men. Despite this limitation, this paper provides important insights that should hopefully be useful in the design of sexual and reproductive health and HIV programmes in Zambia.

Although in this paper the variable on use of alcohol has been observed from the one source only, that is, the 2007 ZDHS, it has been observed that the use of alcohol has been shown to be associated with risky sexual behaviour in various populations (Weiser, Leiter, Heisier, McFarland, Korte, Demonner, *et al.* 2006). The use of alcohol has further been shown to perpetuate the risk of being infected with HIV by suppressing the immune system (Weiser *et al.* 2006). Sexual and reproductive health including HIV and AIDS programmes in Zambia should continue to target young women and men aged 15–24 years and include interventions aimed at dissuading young people from engaging in sexual intercourse while they are intoxicated.

Condom use is one of the most important strategies for controlling the spread of HIV infection. However, on the contrary, it is also thought that educating young people about condoms may promote early sexual initiation (Central Statistical Office, Ministry of Health, Tropical Diseases Research Centre, University of Zambia & Macro International Incorporated 2009). The less pronounced increases in condom use among young men aged 15–24 years as compared to their female counterparts point to a need to strengthen interventions that encourage condom use, particularly among young men. Prevention programmes that support the use of condoms should be invigorated. This assertion is supported by the results from the multivariate analysis which show that the indicator that contributed the most to the observed 2.6% decline in HIV prevalence among young women aged 15–24 in Zambia is the increase in the proportion reporting condom use during premarital sex. Further research geared towards understanding the effectiveness of such programmes in preventing infection from HIV, particularly among young people in Zambia, should be conducted (Slonin-Nevo & Mukura 2005).

The delay of sexual debut among young people has been well embraced in Zambia and should continually be promoted and sustained. Research has shown that the delay in sexual initiation is an indispensable aspect in HIV prevention programmes (Kembo 2012; Ndubani 2002). Promoting abstinence has been an important strategy that has led to the delay in sexual activity among young people in Zambia. Programmes aimed at combating HIV and AIDS in Zambia should deliberately seek to address the higher risk of HIV infection among young women aged 15–24 years relative to their male counterparts.

Furthermore, there is need for comprehensive research to focus on the socio-cultural factors that predispose young men and women in Zambia to higher risk of HIV infection. Such research is crucial in the design of gender-sensitive interventions to control the spread of HIV and AIDS and other sexually transmitted infections and promote sexual and reproductive health, particularly among young people in Zambia. The design of interventions for the control of HIV and AIDS among young people, and indeed in the general population, should take full cognisance of cultural traits and norms and values that sustain risky sexual behaviour and practice.
